# Biogeography of predaceous diving beetles (Coleoptera, Dytiscidae) of Mongolia

**DOI:** 10.3897/zookeys.853.33908

**Published:** 2019-06-06

**Authors:** Davaadorj Enkhnasan, Bazartseren Boldgiv

**Affiliations:** 1 Institute of General and Experimental Biology, Mongolian Academy of Sciences, Peace Avenue 54b, Ulaanbaatar 13330, Mongolia Institute of General and Experimental Biology, Mongolian Academy of Sciences Ulaanbaatar Mongolia; 2 Ecology Group, Department of Biology, National University of Mongolia, Ikh Surguuliin Gudamj 1, Ulaanbaatar 14201, Mongolia National University of Mongolia Ulaanbaatar Mongolia; 3 Academy of Natural Sciences of Drexel University, Philadelphia, PA 19103, USA Academy of Natural Sciences of Drexel University Philadelphia United States of America

**Keywords:** Geographical distribution, altitudinal pattern, dytiscid, range, river sub-basin

## Abstract

The bio-geographical composition and spatial distribution patterns of dytiscid assemblages in Mongolia are relatively unexplored. In this study, we compiled a list of 99 dytiscid species belonging to 20 genera and five subfamilies recorded in Mongolia and investigated species richness, spatial distribution and bio-geographical composition of the Mongolian dytiscid fauna. This study encompasses the information of currently recorded species and their geographic localities in Mongolia based on our own data and literature sources. We examined how dytiscid species richness was related to sub-basins of surface water network, as well as to geographical elevations within Mongolia. The majority of the Mongolian dytiscid fauna was associated with the sub-basins belonging to Arctic Ocean (80 species, 80.8%) and Central Asian Inland (60 species, 60.6%) basins. Only a few species of dytiscids belonged to the remaining river basins. Species richness of dytiscids and total area of sub-basins were not correlated, but species composition of dytiscids differed significantly among the sub-basins.

We observed that most of the species (77 species or 77.8% of total fauna) were recorded in a wide range of elevations and mid-altitudes (1000–2000 m a.s.l.) and showed the greatest diversity of dytiscids. Regarding the bio-geographical composition, species with wide geographical distributions (27.3% of dytiscids), were Palearctic species, while species of Arctic origin (21.2%) together with Boreal elements (16.2%) comprised a large proportion of the dytiscid fauna in Mongolia.

## Introduction

Under global change, natural ecosystems in Mongolia are experiencing greater-than-global average rate of climate change, as well as shifting anthropogenic influences. The country has a large landlocked territory covering an area of 1,564,118 square kilometers in Inner Asia. It is located on the Mongolian Plateau with an average elevation of 1580 m (range of elevation: 560–4374 m), and about 85% of its area lies over 1000 m above sea level (Murzaev 1952).

The country has a large variety of geographic features including high mountains in the west, forests in the north, deserts in the south and plain steppes in the central and eastern regions, with various environmental and geographic formations. Insect biogeographic studies have been done only for a few groups of terrestrial insects of Mongolia (Namkhaidorj 1974; Myagmarsuren 1979, 1996; Bielawski 1984; Puntsagdulam 1994; Bayartogtokh et al. 2014; Buyanjargal et al. 2016). There are no reports of biogeographic studies of aquatic insects, particularly aquatic Coleoptera undertaken in Mongolia. The principal difficulty with any zoogeographical analysis of these groups of insects in Mongolia has been the paucity of taxonomic and distribution data. This situation has improved steadily through the years thanks to the accumulation of more published information such as Brinck (1943), Guéorguiev (1965, 1968a, b, 1969, 1972), Brancucci (1982) and Bellstedt (1985). More recently, some papers by Shaverdo and Fery (2001), Fery (2003) and Shaverdo (2004) have focused on the systematics and taxonomy of dytiscids in Mongolia.

A large number of water beetles were collected in June–July in 2003–2006 and 2008–2011 during the Selenge River Basin and Mongolian Aquatic Insect Survey Expeditions, respectively, as well as in 2009–2015 by Mongolian and Russian researchers within the framework of the Mongolian-Russian Biological Expedition.

Several works on dytiscids have been published based on these studies (Shaverdo and Fery 2006; Enkhnasan 2006, 2008; Shaverdo et al. 2008; Prokin et al. in press), with new faunistic data and new species of dytiscids that were obtained through the expeditions mentioned above. As a result of the latest research efforts on the dytiscid fauna of Mongolia, over 20 species were recorded as new for the country by Shaverdo et al. (2008), and 15 species and two genera by Prokin et al. (in press). Additionally, *Zaitsevhydrus* is recently described in a revision by Fery and Ribera (2018).

Although all of the above-mentioned studies pointed out only general distributions and taxonomy of the dytiscids; a comprehensive overview of the zoogeography of the Mongolian aquatic Coleoptera has not yet been done. In this paper, we attempt to make bio-geographical analyses of dytiscids known for Mongolia, in relation to the country’s surface water network and geographical features.

## Materials and methods

### Study area

Mongolia has an extreme continental climate, with four distinctive seasons. Temperature fluctuates greatly, both daily and annually, with low rainfall (Natsagdorj and Dagvadorj 2010). Average annual temperature ranges between 8.5 °C in the Gobi and -7.8 °C in the high mountains of the Mongolian Altai, Khangai and Khentii ranges. Average annual precipitation is low (200–220 mm) with a range of between 38.4 mm in the Gobi Desert and 389 mm in the North. Seventy percent of Mongolia’s water resources have their source in the Altai, Khangai,and Khentii ranges, the Khuvsgul mountains and the higher part of Ikh Khyngan range, which covers 30% of the Mongolian territory (Natsagdorj 2014). Surface water resources in Mongolia are limited, unevenly distributed (Batnasan 2003) and also highly vulnerable to climatic conditions.

**Figure 1.**
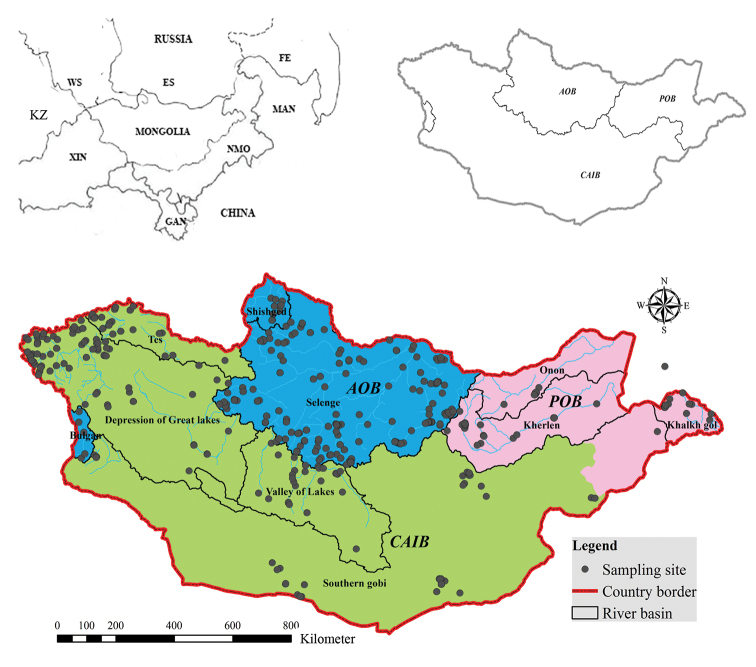
Watersheds and sub-basins of Mongolia. Gray dots represent all sampling points of dytiscids. Abbreviations: KZ – Kazakhstan; WS – West Siberia; ES – East Siberia; FE – Far East; MAN – Manchuria; NMO – Nei Mongol; GAN – Gansu; XIN – Xinjiang; AOB–Arctic Ocean Basin; POB – Pacific Ocean Basin; CAIB – Central Asian Inland Basin.

Our database consists of dytiscid samplings from ten sub-basins belonging to three water basins. Mongolia is situated on three international river basins (Davaa and Jambaljav 2014): the Arctic Ocean Basin (AOB), Pacific Ocean Basin (POB) and Central Asian Internal Drainage Basin (CAIB), which are subdivided into 10 regional basins. Three of these sub-basins, Selenge (SRB), Shishkhed (ShRB) and Bulgan (BRB) are included in the Arctic Ocean Basin; three sub-basins, Kherlen (KhRB), Onon (ORB) and Khalkh gol (KhGRB) belong to Pacific Ocean Basin; while four sub-basins, namely Tes (TRB), Depression of Great Lakes (DGLB), Valley of Lakes (VLRB) and Gobi (GRB) are in the Central Asian Inland Basin (Fig. 1).

Rivers belonging to AOB are most extensively developed in the north and constitute the country’s major river system, i.e., the Selenge River system, which drains via Lake Baikal to the Arctic Ocean. Some tributaries of Siberia’s Yenisei River, which also flows to the Arctic Ocean, originate in the mountains of northwestern Mongolia (Davaa 2015).

Many rivers of western Mongolia end at lakes in the CAIB, most often in those of the Great Lakes Depression. The depression is named so because it contains six major Mongolian lakes: the saline Uvs Nuur, Khyargas Nuur, Durgun Nuur and the fresh water Khar-us Nuur, Khar Nuur and Airag Nuur, as well as number smaller ones. The major rivers are Khovd, Zavkhan, and Tes. The few streams of southern Mongolia also do not reach the sea but run into lakes or deserts (Davaa 2015).

In northeastern Mongolia, rivers of POB such as Onon, Kherlen and Khalkh gol River drain into the Pacific after joining the Shilka River in Russia and the Amur (Heilong Jiang) rivers, forming the tenth longest river system in the world (Davaa 2015).

There are about 4113 rivers in Mongolia, with a total length of 67,000 km. The longest river is the Orkhon at 1124 kilometers in length. Large rivers originate in the mountainous areas in the north and west of the country – primarily in the Mongol Altai, Khangai-Khuvsgul and Khentii mountain ranges – where small rivers and mountain streams merge to create well-developed water networks. There are also over 3000 big and small lakes, 6900 springs, 190 glaciers and 250 mineral water springs in the country (Davaa 2015).

The surface water network is of greatest density in the north of the country. In contrast, the southern, central and southeastern parts of the country have few rivers or other surface water resources. In the interior drainage basins, in the western and southern areas of Mongolia, seasonal or intermittent streams end in salt lakes or disappear into the desert. The rivers’ main water sources are rainfall, groundwater, snow and glaciers, with melting snow accounting for 15–20 percent of the annual runoff (Davaa and Jambaljav 2014).

### Data collection

During our study, we collected 3517 beetle specimens from 630 sampling points (Fig. 1). In addition, we complemented our own data with all previously available information on dytiscid species in Mongolia. Sampled sites covered the main habitats in all water sub-basins of Mongolia, though the number of samples in each region was different because of their different area, habitat types and remoteness (see Figure 1). The material included in our compilation was collected from different regions of Mongolia by many researchers. In total, dytiscids were collected from 1077 sampling points, which involved 6122 specimens. In this paper, we include only those specimens for which collection localities were clearly reported. This selection was necessary, because some records reported by other researchers from across Mongolia did not have clearly identifiable sampling sites. All species names were updated according to the latest catalogue of Palearctic Coleoptera (Nilsson and Hájek 2018). Generic and species names of dytiscids are listed in taxonomical order (Appendix 1).

The relative area of the water sub-basin of Mongolia was taken from the classification of Mongolian water resources (Davaa 2015). Bio-geographical analysis of dytiscid species found in Mongolia was based on our own data and literature sources that provide information on geographical ranges (Nilsson and Hájek 2018).

Predaceous diving beetle collections are currently deposited in the Laboratory of Entomology, Institute of General and Experimental Biology, Ulaanbaatar, Mongolia.

We have classified the dytiscid species of Mongolia in accordance with the earlier systematic work of Zaitsev (1972). The range of some species in Mongolia has not been exactly determined yet. The range patterns currently recognized are:

Palearctic. Species distributed throughout the whole Palearctic Region.

Holarctic. Inhabitants of the northern regions of the European Russia and Siberia (to Kamchatka i.e., tundra and taiga).

Oriental. Occurring at the border between Palearctic and Oriental regions: India and Pakistan, Kashmir, Himachal Pradesh, Uttar Pradesh, Nepal, Sikkim, and Darjeeling, Bhutan, Arunachal Pradesh.

Arctic. Species of Arctic origin with occurrence in the tundra and the northern edge of the taiga. They are distributed in the south to Transbaikalia and in Western Europe to northern Sweden and Norway.

Boreal. This group is the largest. They live in the taiga and insular forests of Eurasia, in the plains. They occur in the northern and central belt of European Russia, in Siberia and highlands of the Caucasus; they also occur in northern and central Europe to eastern France and northern Italy.

Mediterranean. Species widely distributed throughout the Mediterranean (southern Europe, North Africa, and Asia Minor); east Mediterranean species occur in the Balkans, Asia Minor, Syria and western Iran.

Steppe. Species of the steppe zone of European Russia, western Siberia, northern Kazakhstan, eastern Transcaucasia, Turkmenia (some species of this group reach Hungary and Austria in the west).

Turanian. Species occurring in the mountains of central Asia, Sinkiang, Tien Shan.

Palearchearctic. Species from Korea, China and Japan.

### Data analysis

Similarities of dytiscid assemblages among the sub-basins were calculated using the Bray-Curtis’ quantitative formula (Bray and Curtis 1957) and the Simple Average Linkage for hierarchical clustering of objects. The results obtained were presented in a similarity dendrogram. Similarities among objects were determined using Biodiversity Pro v.2 software (McAleece et al. 1997). Square-root transformation was used to meet the assumption of normality because the data were counts (Sokal and Rohlf 2012). The occurrences of dytiscids at different altitudes and water sub-basins were arranged in presence/absence tables. Pearson’s product-moment correlation was used to determine the relationships between area sizes of each sub-basin and their species richness. The differences in dytiscid fauna among the sub-basins were clarified using one-way analysis of variance (ANOVA). All statistical analyses were performed with software R 3.1.3 for Windows (Team 2015). For all statistical tests, we considered results significant when *p* < 0.05.

## Results

### Diversity of dytiscids

Based on our investigations, the dytiscid fauna of Mongolia comprises 99 species belonging to 20 genera in five subfamilies. A list of dytiscid species and their occurrences in various sub-basins of Mongolia are given in Appendix 1. It is necessary to note that the sub-basins of Khalkh gol, Bulgan and Gobi region are still insufficiently investigated.

The greatest diversity of dytiscids was recorded from the Selenge River Basin and Depression of Great Lakes. The majority of dytiscid species of Mongolia was represented by two subfamilies, Hydroporinae (44 species) and Agabinae (36), that altogether comprise about 80 percent of the total dytiscid species. The other three subfamilies were Dytiscinae (11), Colymbetinae (7) and Laccophilinae (1), which were only represented by a few species in Mongolia (Appendix 1).

The most species-rich and commonly encountered genera in Mongolia were *Agabus* (25 species), *Hygrotus* (14), *Hydroporus* (14), *Ilybius* (10), *Nebrioporus* (4), *Graphoderus* (4) and *Rhantus* (4). The other genera included less than four species each. *Colymbetes*, *Dytiscus*, *Hydroglyphus* and *Oreodytes* were each represented by three species, while *Hydaticus*, *Acilius* and *Bidessus* had two species each. Six genera were represented by a single species, namely *Boreonectes*, *Laccophilus*, *Laccornis*, *Nectoporus*, *Platambus* and *Zaitsevhydrus*. Thus, few genera were species-rich, whereas the majority comprised fewer species, with the mean number per genus = 5.9 species.

**Figure 2.**
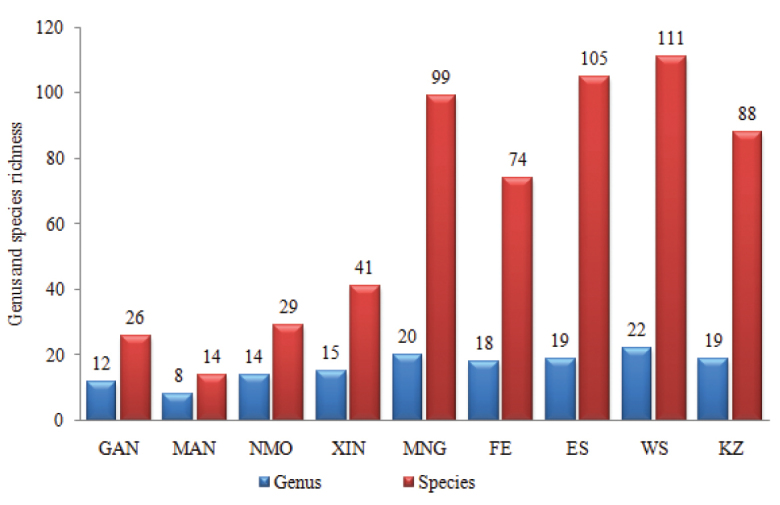
Generic and species richness of dytiscids in Mongolia and its surrounding regions. Abbreviations: GAN – Gansu; MAN – Manchuria; NMO – Nei Mongol; XIN – Xinjiang; MNG – Mongolia; FE-Russian Far East; ES – East Siberia; WS – West Siberia; KZ – Kazakhstan. Source: Catalogue of Palearctic Coleoptera (Nilsson and Hájek 2018).

Because China and Russia are large countries and have many diverse zones geographically, our analysis also focused on species composition of surrounding regions in these countries adjacent to Mongolia, in order to reveal species which are shared among them. Fauna of dytiscids in the closest seven regions of Russia and China, as well as Kazakhstan were included. Based on information of the distribution of 261 species, 27 genera of dytiscids were compiled from adjacent regions and Mongolia and a presence or absence matrix for species in these regions was constructed. These analyses found that faunistic similarity coefficients between Mongolia and adjacent regions ranged from 7.1% to 58.4% (Fig. 2, Table 1).

**Table 1.** Similarity index of the Mongolian dytiscid fauna with neighboring regions.

	GAN	MAN	NMO	XIN	FE	ES	WS	KZ
MNG	13.0	7.1	18.9	15.9	45.3	58.4	49.0	39.8
GAN	*	0.0	25.9	24.6	10.1	10.9	4.4	10.6
MAN	*	*	4.7	7.4	9.1	6.8	9.7	3.9
NMO	*	*	*	20.3	25.2	18.0	11.5	10.3
XIN	*	*	*	*	10.5	8.3	9.3	28.1
FE	*	*	*	*	*	48.3	33.7	22.2
ES	*	*	*	*	*	*	67.3	45.8
WS	*	*	*	*	*	*	*	54.5

Most of the species recorded for the dytiscid fauna of Mongolia (86 species) were common with those of the surrounding regions. The East (similarity index 58.4%, 60 species shared) and West Siberia (51 species shared) regions of Russia showed the closest similarity with the Mongolian dytiscid fauna. The Manchuria (7.1%, four species shared) region of China had the lowest faunal similarity with Mongolia (Fig. 2). Hence, Mongolian dytiscid fauna has a much closer similarity with that of the northern (Russia) than the southern regions (China).

### Geographical distribution of dytiscids by water sub-basins

Dytiscids in the 10 sub-basins occurred with various numbers of species, from 5 to 79. Each of these sub-basins had a peculiar composition of dytiscids, but there were several dominant species in most of the sub-basins. Seventy-nine species were found from Selenge River Basin, 45 species from the Depression of Great Lakes, 26 species from Gobi Basin, 22 species from Tes River Basin and 21 species from Shishkhed and Valley of Lakes River Basins. The species richness of dytiscids in other sub-basins varied between 5 and 17 (see Appendix 1).

Only *Hygrotusmarklini* Gyllenhal, 1813 was common to nine sub-basins, six other species, namely *H.impressopunctatus* Schaller, 1783, *H.flaviventris* Motschulsky, 1860 *Agabusadpressus* Aubé, 1837, *Laccophilusbiguttatus*, *Oreodytesmongolicus* Brinck, 1943 and *Rhantusfrontalis* Marsham, 1802 inhabited seven out of 10 sub-basins, which provide a variety of habitats. While other five species, *A.coxalis* Sharp, 1880, *R.notaticollis* Aubé, 1837, *Dytiscusdauricus* Gebler, 1832, *Nebrioporusairumlus* Kolenati, 1845 and *Hydroporusacutangulus* complex Thomson, 1856 were found in six sub-basins. *Agabusdichrous* Sharp, 1878, *Hygrotusunguicularis* Crotch, 1874, *Ilybiuspoppiusi* Zaitzev, 1907, *Nectoporussanmarkii* Sahlberg, 1826 and *O.septentrionalis* Gyllenhal in C.R. Sahlberg, 1826 were recorded from five sub-basins, while nine other species, *A.infuscatus* Aubé, 1838, *A.pallens* Poppius, 1905, *Graphoderusaustriacus* Sturm, 1834, *H.enneagrammus* Ahrens, 1833, *H.nigrolineatus* Steven in Schönherr, 1808, *I.cinctus* Sharp, 1878 and *I.lateralis* Gebler, 1832 were distributed in four sub-basins. The other 32 species were found in two or three sub-basins. From our data it is apparent that 43 species have restricted ranges within Mongolia, because of their distribution being restricted to a single sub-basin (see Appendix 1).

**Figure 3.**
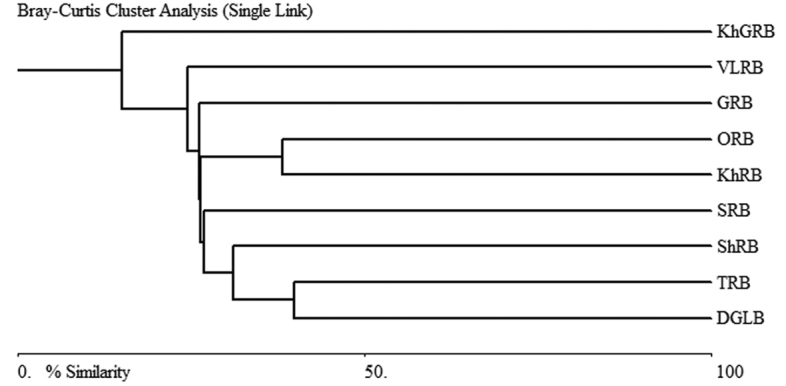
A dendrogram depicting the dytiscid faunal similarity among the water sub-basins of Mongolia. DGLB – Depression of Great Lakes Basin; GRB – Gobi River Basin; TRB – Tes River Basin; VLRB – Valley of Lakes River Basin; KhGRB – Khalkh Gol River Basin; KhRB – Kherlen River Basin; ORB – Orkhon River Basin; SRB – Selenge River Basin; ShRB – Shishkhed River Basin; BRB – Bulgan River Basin.

Based on the information of the distribution of 99 dytiscid species from different river basins in Mongolia, a presence or absence matrix for species in the nine regions was constructed, except BRB because there were only five species recorded so far (See Appendix 1). Differences between the faunal compositions of dytiscids in the various sub-basins were as theoretically expected. Most of these sub-basins that are more similar in dytiscid fauna were geographically adjacent to one another (KhGRB with ORB, 38.2%; DGLB with TRB, 39.8%), and reflected the main landscape pattern of Mongolia. The relatively low similarity was observed between distant and ecologically different sub-basins, such as SRB with KhGRB (1.3) and VLRB (3.7%); DGLB with KhGRB (4.4%) (Fig. 3).

It should be noted that because of different sampling effort, the diversity of dytiscids reported here for some basins, such as the Valley of Lakes, Bulgan River Basin and Gobi Basin etc. might not be fully representative of reality. Overall, the dytiscid fauna of the various basins was relatively distinct, confirming the well-established classification of the sub-basins in Mongolia.

**Table 2.** Numbers of dytiscid species of different zoogeographical origins listed for 10 sub-basins of Mongolia.

Bio-geographical ranges	AOB			CAIB				POB		
	SRB	ShRB	BRB	DGLB	GRB	TRB	VLRB	KhGRB	KhRB	ORB
Arctic origin	19	7	1	13	5	4	6	2	5	4
Boreal	13	5	2	9	3	5	4	2	3	5
Holarctic	1	1	0	1	0	1	2	0	0	0
Turanian	5	3	0	4	3	2	2	0	1	1
Mediterranean	0	0	0	0	2	1	0	0	0	0
Oriental	0	0	0	0	0	0	0	0	0	0
Palearchearctic	2	0	0	2	1	0	1	0	1	1
Palearctic	32	4	1	10	7	7	5	0	4	2
Steppe zone	7	1	1	6	5	4	1	5	3	2
**Overall species**	**79**	**21**	**5**	**45**	**26**	**23**	**21**	**9**	**17**	**16**

Abbreviations: AOB-Arctic Ocean Basin: SRB-Selenge River Basin, ShRB-Shishkhed River Basin, BRB-Bulgan River Basin. CAIB-Central Asian Inland Basin: DGLB- Depression of Great Lakes Basin, GRB-Gobi River Basin, TRB-Tes River Basin, VLRB-Valley of Lakes River Basin. POB-Pacific Ocean Basin: KhGRB-Khalkh Gol River Basin, KhRB-Kherlen River Basin, ORB-Orkhon River Basin.

### Species-area relations

Given the geographic distribution in various sub-basins of Mongolia reported above, there were some mismatches between the area of each basin and the respective number of dytiscid species. The highest number of species (79 of a total of 99 species) was recorded in the Selenge River Basin, though the total area of this region is only 18.9% of the territory of Mongolia. Forty-five species were found from the Depression of Great Lakes that covers 16.0% of the country. Twenty-one and twenty-six species were recorded in Shishkhed and Gobi basins, which comprise 1.3% and 39.9 % of the entire area of the country, respectively. Species richness of dytiscids varied also among the sub-basins. One-way ANOVA showed that significant differences were observed in the species richness (*F*_9, 1077_ = 4.34; *p<*0.0001) among the sub-basins. However, there was no significant relationship between the species richness of dytiscids and the total area of each sub-basin (*r* = 0.46, *p* = 0.1708).

### Altitudinal patterns of diversity

**Figure 4.**
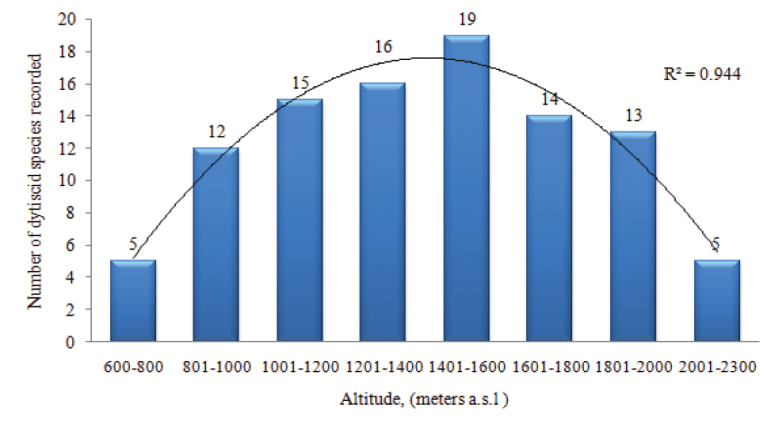
Altitudinal ranges of dytiscids in Mongolia, showing number of species in each category of altitudinal range, with a minimum range of 600 m and a maximum of 2300 m.

Our analysis of patterns of dytiscid species distribution with altitudes was based upon collection data from 1077 different elevation points. Dytiscid community composition was different by altitudes, as most of the species were recorded in a wide range of elevations (Fig. 4).

The number of species observed in the various altitude ranges differed significantly (*p* = 0.0006). Twelve species, namely *Aciliussulcatus* Nicolai, 1822, *Agabuskaszabi* Guéorguiev, 1972, *A.moestus* Curtis, 1835, *Bidessusnasutus* Sharp, 1887, *Colymbetespseudostriatus* Nilsson, 2002, *Hydaticusaruspex* Clark, 1864, *Hygrotusinaequalis* Fabricius, 1777, *Hydroporuspalustris* Linnaeus, 1760, *Ilybiuscinctus* Sharp, 1878, *Ilybiuserichsoni* Gemminger & Harold, 1868, *Laccornisoblongus* Stephens, 1835, *Platambusmaculatus* Linnaeus, 1753 occurred in the range of 801–1000 m a.s.l., while *Graphoderuszonatusverrucifer* Sahlberg, 1824, *Hydroporusangusi* Nilsson, 1990, *H.fuscipennis* Schaum & Kiesenwetter, 1867, *Ilybiusbalkei* Fery & Nilsson, 1993 and *I.opacus* Aubé, 1837 were restricted to lower altitudes, between 600 and 800 m a.s.l..

Several other species (i.e. *Agabuscostulatus* Motschulsky, 1859, *A.lineatus* Gebler, 1848, Boreonectesaff.emmerichi Falkenström, 1936, *Hydroporusmorio* Aubé, 1838 and *H.notabilis* LeConte, 1850) were reported at elevations of 2001 – 2300 m a.s.l..

Species richness was greatest between 1000 and 2000 m a.s.l., as 75 species (77.8% of total diversity) was recorded in this mid-altitude range. Thus, we found a peak of species richness between 1400 and 1600 m a.s.l.

### Geographic distribution of species in Mongolia

**Figure 5.**
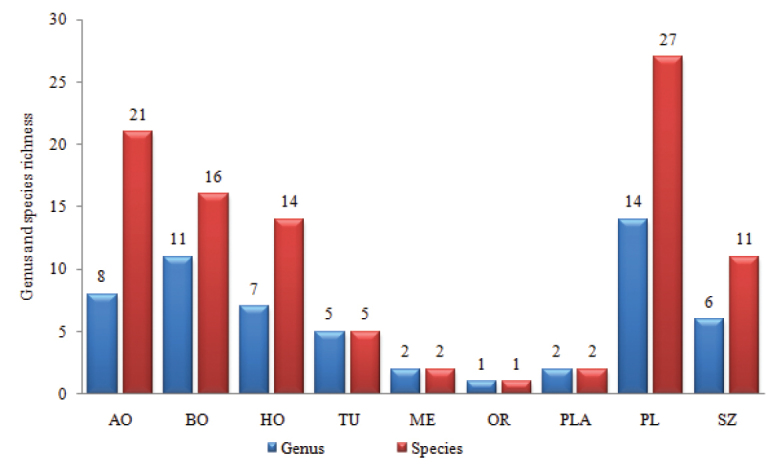
Relationship of generic and species diversity of dytiscids in the water sub-basins of Mongolia according to their biogeographic regions. Abbreviations: AO – species of Arctic origin; BO – Boreal species; HO – Holarctic species; TU – Turanian species; ME – Mediterranean elements; OR – Oriental; PLA – Palearchearctic; PL – species distributed throughout the whole Palearctic; SZ – species of the Steppe zone.

The geographic distribution of all known species of dytiscids in Mongolia was compiled, and the species were divided into groups based upon their range. Species of Boreal (16 species, 16.8%) and Arctic origin (21 species, 22.1%) comprised a large proportion of the dytiscid fauna, due to the extremely harsh and fluctuating climate of Mongolia (Fig. 5).

Two species, *Oreodytesmongolicus* and *Agabuskaszabi* were found to be endemic to Mongolia. Although *O.mongolicus* was common throughout the whole country, it was collected mainly from sub-basins DGLB, TRB and SRB. Twenty-seven were Palearctic species (27.3% of total), namely *Aciliussulcatus*, *Agabusblatta*, *A.congener*, *Agabuskaszabi*, *A.kholini*, *A.laferi*, *Agabussvenhedini*, *A.udege*, *Bidessusunistriatus*, *Boreonectesaff.emmerichi*, *Colymbetespseudostriatus*, *Graphoderuscinereus*, *Hydaticuscontinentalis*, *Hydroglyphuslicenti*, *Hydroporusangusi*, *H.crinitisternus*, *H.kabakovi*, *H.palustris*, *H.uenoi*, *Hygrotuschinensis*, *H.inaequalis*, *H.parallellogrammus*, *Ilybiusbalkei*, *I.chishimanus*, *Oreodytesshorti*, *Platambusmaculatus* and *Rhantusvermiculatus*. Twenty-one were Arctic species (22.1%), namely *Agabusadpressus*, *A.aequalis*, *A.arcticusalpinus*, *A.costulatus*, *A.coxalis*, *A.lapponicus*, *A.moestus*, *A.thomsoni*, *Colymbetesdahuricus*, *C.dolabratus*, *Dytiscuslatro*, *Hydroglyphushamulatus*, *Hydroporusacutangulus* complex, *H.sibiricus*, *H.submuticus*, *Hygrotusunguicularis*, *Ilybiuslateralis*, *I.obtusus*, *I.poppiusi* and *Oreodytesmongolicus*. Sixteen Boreal species (16.1%), such as *Aciliuscanaliculatus*, *Agabusbiguttulus*, *A.discolor*, *Graphoderuszonatusverrucifer*, *G.zonatuszonatus*, *Hydroglyphusgeminus*, *Hydroporuselongatulus*, *Hygrotusmarklini*, *H.quinquelineatus*, *Ilybiusangustior*, *Laccornisoblongus*, *Nebrioporusassimilis*, *N.depressus*, *Oreodytesseptentrionalis*, *Rhantusfrontalis*, and *R.notaticollis* were also found to be widely distributed in Mongolia.

The quantitatively most important bio-geographical elements were Holarctic (14 species, 14.1%), Steppe (11 species, 11.1%) and Turanian (5 species, 5.0%) species. Mediterranean (*Hygrotusconfluens* and *Agabusnebulosus*) and Palearctic species (*Dytiscusdauricus*, *Nebrioporushostiles*) were each represented by two species. Finally, only one species from the Oriental Region (*Agabusjaponicuscontinentalis*) was recorded from Mongolia.

## Discussion

### Species richness

Mongolia has representatives of about 1.12% of the known world dytiscid genera and 2.3% of the currently described species (Nilsson 2015; Nilsson and Hájek 2018). Patterns of climatic and environmental conditions might be the main factor controlling dytiscid fauna in Mongolia and surrounding regions. Species composition of dytiscids in the surrounding countries and regions is similar to that of Mongolia, with Russian Far East having 74 species, East Siberia with 105, West Siberia with 111, Kazakhstan with 88 and China (altogether, including Gansu, Manchuria, Inner Mongolia and Xinjiang) with 110 species recorded respectively (Nilsson and Hájek 2018).

The Agabinae and Hydroporinae are the largest dytiscid subfamilies in the world and the dominant groups in most habitats. The prevalence of these subfamilies has been reported to increase with increasing variety of water bodies (Lawrence and Slipinski 2013; Yee 2014) . The faunal composition of dytiscids in Mongolia was also consistent with this pattern: species belonging to the Agabinae and Hydroporinae comprised more than 80% of the total number of species recorded in this study, while Dytiscinae and Colymbetinae were represented by eleven and seven species, respectively, with a single species of Laccophilinae also being recorded.

More than half of the recorded species in the Mongolian dytiscid fauna belong to the genera *Agabus*, *Hygrotus*, *Hydroporus* and *Ilybius*. Other genera containing three or more species in Mongolia were: *Dytiscus* (3 species), *Graphoderus* (4), *Nebrioporus* (4), *Oreodytes* (3), *Rhantus* (4), *Colymbetes* (3) and *Hydroglyphus* (3). Together, the eleven most diverse genera constituted nearly 88.5% of the dytiscid species known from Mongolia, while other genera, such as *Acilius*, *Hydaticus*, *Bidessus*, *Boreonectes*, *Laccophilus*, *Laccornis*, *Nectoporus*, *Platambus* and *Zaitsevhydrus* comprised a much smaller proportion of the fauna. Similar faunistic patterns were found in other regions surrounding Mongolia, e.g., Russian Far East and northern China (Jäch and Ji 1998; Nilsson and Hájek 2018). Jäch and Ji (2003) reported 31 species belonging to 13 genera from Xinjiang (China); one species from Ningxia; 16 species (nine genera) from Gansu; 10 species (eight genera) from Shanxi, and eight species (six genera) from Chinese Manchuria. Enkhnasan (2018) recorded 36 species, 16 genera from Inner Mongolia based on the collection of the Nonnaizab Entomology Center, Normal University, Inner Mongolia, as well as literature sources such as Jäch and Ji (1995; 1998; 2003), Morse et al. (1994) and Nonnaizab (1999).

Shaverdo et al. (2008) recorded 87 (without subspecies) species belonging to 15 genera from Mongolia. Among them, nine species did not include accurate geographic locality, only a distribution range given as “Mongolia” (Nilsson 2003; Shaverdo et al. 2008; Nilsson and Hájek 2018). Those species were *Agabusbasalis* (Gebler, 1829), *A.brandti* Harold, 1880, *A.confinis* (Gyllenhal, 1808), *A.fulvaster* Zaitsev, 1906, *Ilybius f. fuliginosus* (Fabricius, 1792), *Rhantusbistriatus* (Bergstrasser, 1777), *R.suturalis* (Macleay, 1825), *Cybistertripunctatuslateralis* (Fabricius, 1798) and *Laccophilusminutus* (Linnaeus, 1758). Therefore, in our analysis we included only species with specific geographic locations in Mongolia collected by other researchers, while excluding the nine species above. Perhaps, these species might be confirmed in future studies for Mongolia. Prokin et al. (in press) newly recorded two genera and 15 species for the country. Also, we recorded *Agabusudege* Nilsson, 1994 and *Agabusnebulosus* Forster, 1771 as new for the country and compiled from other previous records another three species, including *Aciliuscanaliculatus* Nicolai, 1822, *Bidessusunistriatus* Goeze, 1777, *Nebrioporushostilis* Sharp, 1884 (Guéorguiev 1972; Enkhnasan 2006; Prokin and Zhavoronkova 2015). In total, there were 99 species belonging to 20 genera of dytiscids recorded for Mongolia. Sampling points for 23 of these species were derived from the literature (Appendix 1).

Calosi et al. (2010) indicated that absolute thermal tolerance range is the best predictor of species’ latitudinal range extent and position, while differences in dispersal ability (based on wing size) apparently are less important for European diving beetle species, with the northern and southern range limits related to their tolerance of low and high temperatures, respectively. In general, dytiscid species richness depends on the altitude and water network of the country considered. The most favoured altitude for dytiscids in Mongolia was in the range of 1400–1600 m a.s.l.; at lower or higher altitudes species richness of dytiscids decreased steadily.

### Distribution in sub-basins

The “Arctic Ocean Basin” group encompasses the Selenge, Shishkhed and Bulgan River Basins. The “Pacific Ocean Basin” group includes the Kherlen, Onon and Khalkh Gol River Basin. The “Central Asian Inland Basin” group consists of the Tes, Depression of Great Lakes, Valley of Lakes and Gobi Basin. The results show that the faunal composition of dytiscids is more similar among the sub-basins due to geographical adjacency to one another, and reflect the main landscape pattern of Mongolia. In particular, the similarity of dytiscid fauna between AOB and CAIB was 36.5% and between CAIB and POB 28.6%.

It is notable that the most common species (e.g. *Hygrotusimpressopunctatus*, *H.marklini*, *Agabusadpressus*, *A.coxalis*, *Oreodytesmongolicus*, *O.septentrionalis*, *Rhantusnotaticollis*, *Dytiscusdauricus*, *Hydroporusacutangulus* complex, *Hygrotusflaviventris*, *Laccophilusbiguttatus*) tended to be widely distributed across various sub-basins, but in contrast the uncommon and rare species (*Agabusbiguttulus*, *A.clavicornis*, *A.congener*, *A.angusi*, *A.kholini*, *A.laferi*, *A.lapponicus*, *A.lineatus*, *Colymbetesdahuricus*, *C.pseudostriatus*, *Graphoderuscinereus*, *G.zonatusverrucifer*, *Hydroglyphuslicenti*, *Hydroporusangusi*, *H.elongatulus*, *H.fuscipennis*, *H.kabakovi*, *H.morio*, *H.nigellus*, *H.notabilis*, *H.palustris*, *H.submuticus*, *H.uenoi*, *Hygrotusflaviventris*, *H.urgensis*, *I.balkei*, *I.chishimanus*, *I.erichsoni*, *I.opacus* and *Platambusmaculatus*) were highly restricted in their distribution, generally occurring only in one basin.

Aquatic macro-invertebrate assemblages can be affected by various local and regional environmental factors, such as chemical and physical characteristics of stream water, hydrology, and geographic location, as well as climatic factors. Hayford and Gelhaus (2010) concluded that water temperature, pH, conductivity and elevation were not significant predictors of variation in aquatic insect metrics for Mongolian surface waters, but diversity in some families of aquatic insects tended to increase with increased erosion, conductivity, and pH, according to the large-scale Mongolian Aquatic Insect Survey results.

Generally, the species richness of local dytiscid communities is primarily influenced by climatic conditions (e.g. temperature regimes, precipitation), landform, and microhabitat patterns (e.g. vegetation cover, erosion, variety of water bodies). In Mongolia, however, due to the high habitat heterogeneity, species diversity of dytiscids in various sub-basins may differ as a consequence of water physico-chemical parameters that can determine whether a species is present or absent within a locality; thus, small-scale patterns of habitat distribution are important for dytiscids in Mongolia, especially in arid regions.

### Altitudinal distribution

We suggest that the clear differentiation of elevational distribution observed for the majority of dytiscid species considered in this study is due to local geographic relief, as the majority of the country exhibits mountainous landscapes (about 85% of its area is over 1000 m a.s.l.), the exception being the plain grasslands of eastern Mongolia.

The mid-elevation peak in dytiscid diversity is sometimes attributed to the warmer and better wetland habitat conditions and prey resource availability at these elevations. Based on research from various regions, it has been observed that high diversity of diving beetles depends on the number of wetland types represented in a landscape, and thus it is possible to achieve high diversity in a small area by combining permanent and temporary wetlands, as well as systems located in wooded and open environments (Lundkvist et al. 2001; Bloechl et al. 2010; Mabidi et al. 2017). The absence of dytiscid species at elevations above 2300 m a.s.l. might be due to low temperature and limited water sources, both of which would preclude their distribution at high altitudes in Mongolia. It is worth noting that this is the first report on the elevational distribution of dytiscids in Mongolia, and the distinct pattern of dytiscid distribution in various elevation ranges might also be caused by different degrees of sampling effort applied during investigations in different basins of the country.

### Biogeography

The biogeographic composition of dytiscid fauna in Mongolia confirms that it is one of the representative parts of the Palearctic dytiscid fauna. The majority of dytiscid species in Mongolia are widespread in the whole Palearctic region, with the addition of Holarctic elements. Thus, species of Arctic and Boreal regions are widely distributed in Mongolia and comprise more than one third of the total number of species (37.4%). The other specific characteristic of the Mongolian dytiscid fauna is the presence of species from the Oriental Region, as for instance *Agabusjaponicuscontinentalis*.

Finally, it should be noted that the magnitude of climatic changes in temperature and precipitation are predicted to stress a variety of ecosystems directly or indirectly. Most attention has focused on how climate change will affect terrestrial ecosystems, but aquatic ecosystems (e.g., ponds, lakes, streams, and rivers) will also experience parallel changes in diel, seasonal, and annual temperature and precipitation patterns. Therefore, we need to focus on issues related to the effect of increased temperature on the characteristics of biogeographical distribution of dytiscids. Detailed biogeographical surveys play an important role in providing information of what species are present in sub-basins and understanding their ecological roles, to better manage and protect aquatic ecosystems for the future.

## Appendix 1

**Table:** Compiled dytiscid species list from own data and literature sources, and their distribution in sub-basins of Mongolia.

ID	Species name	DGLB	GRB	TRB	VLRB	KhGRB	KhRB	ORB	SRB	ShRB	BRB	Total occurrence in the basins	Total abundance of species
** Agabinae **													
1	*Agabusaequalis* Sharp, 1882	0	0	0	0	0	0	0	1	0	0	1	3
2	**Agabusamoenus* Solsky, 1874	0	1	0	0	0	0	0	0	0	0	1	5
3	**Agabusangusi* Harris, 1828.	0	0	0	0	0	0	1	0	0	0	1	1
4	*Agabusarcticusalpinus* Motschulsky, 1860	1	0	0	0	0	0	0	1	1	0	3	21
5	*Agabuscongener* Thunberg, 1794	0	0	0	0	0	0	0	1	0	0	1	1
6	*Agabuscostulatus* Motschulsky, 1859	1	0	0	0	0	0	0	1	0	0	2	24
7	*Agabuscoxalis* Sharp, 1880	1	1	0	0	1	0	1	1	1	0	6	21
8	*Agabusinfuscatus* Aubé, 1838	1	0	0	1	0	0	0	1	1	0	4	18
9	**Agabusjaponicuscontinentalis* Guéorguiev, 1970	0	1	0	0	0	0	0	0	0	0	1	3
10	**Agabuskaszabi* Guéorguiev, 1972	0	0	0	0	0	0	1	0	0	0	1	1
11	*Agabuslapponicus* Thomson, 1867	0	0	0	0	0	0	0	1	0	0	1	6
12	**Agabusmoestus* Curtis, 1835	0	0	0	0	0	0	0	1	0	0	1	1
13	**Agabusthomsoni* Sahlberg, 1871	0	0	0	0	0	0	0	1	0	0	1	2
14	*Agabusclavicornis* Sharp, 1882	1	0	0	0	0	0	0	1	0	0	2	9
15	*Agabuslineatus* Gebler, 1848	0	0	0	0	0	0	0	1	0	0	1	1
16	*Agabuspallens* Poppius, 1905	1	0	1	0	0	0	1	1	0	0	4	17
17	*Agabusadpressus* Aubé, 1837	1	1	1	1	0	1	0	1	1	0	7	58
18	**Agabusbiguttulus* Thomson, 1867	0	0	0	0	0	0	1	0	0	0	1	1
19	**Agabusblatta* Jakovlev, 1897	1	0	1	0	0	0	0	0	0	0	2	2
20	*Agabusdichrous* Sharp, 1878	1	1	1	1	0	0	0	1	0	0	5	40
21	*Agabuskholini* Nilsson, 1994	0	0	0	0	0	1	0	0	0	0	1	1
22	*Agabuslaferi* Nilsson, 1994	0	0	0	0	0	0	0	1	0	0	1	2
23	*Agabusnebulosus* Forster, 1771	0	1	1	0	0	0	0	0	0	0	2	2
24	**Agabussvenhedini* Falkenström, 1932	1	0	1	0	0	0	0	0	0	0	2	2
25	*Agabusudege* Nilsson, 1994	0	0	0	1	0	1	0	1	0	0	3	4
26	*Ilybiusangustior* Gyllenhal, 1808	1	0	0	0	0	0	0	1	0	0	2	12
27	*Ilybiusbalkei* Fery&Nilsson, 1993	0	0	0	0	0	0	0	1	0	0	1	1
28	*Ilybiuschishimanus* Kôno, 1944	0	0	0	0	0	0	0	1	0	0	1	9
29	*Ilybiuscinctus* Sharp, 1878	0	1	0	0	1	1	1	0	0	0	4	8
30	*Ilybiuserichsoni* Gemminger & Harold, 1868	0	0	0	0	0	0	0	1	0	0	1	4
31	*Ilybiuslateralis* Gebler, 1832	1	0	0	0	0	0	1	1	1	0	4	8
32	*Ilybiusobtusus* Sharp, 1882	0	0	0	1	0	0	0	1	1	0	3	10
33	*Ilybiusopacus* Aubé, 1837	0	0	0	0	0	0	0	1	0	0	1	2
34	*Ilybiuspoppiusi* Zaitzev, 1907	1	1	0	0	1	0	1	1	0	0	5	26
35	*Ilybiussubaeneus* Erichson, 1837	1	0	0	0	0	0	0	1	0	0	2	5
36	*Platambusmaculatus* Linnaeus, 1753	0	0	1	0	0	0	0	0	0	0	1	1
** Colymbetinae **													
37	*Colymbetesdahuricus* Aubé, 1837	0	0	0	1	0	0	0	1	0	0	2	8
38	**Colymbetesdolabratus* Paykull, 1798	1	0	0	1	0	0	0	0	0	0	2	4
39	*Colymbetespseudostriatus* * Nilsson, 2002	0	0	0	0	0	0	0	1	0	0	1	1
40	*Rhantusfrontalis* Marsham, 1802	1	1	1	1	1	0	1	1	0	0	7	16
41	*Rhantusnotaticollis* Aubé, 1837	1	0	1	0	0	1	1	1	1	0	6	62
42	**Rhantusrufus* Zimmermann, 1922	0	0	0	0	0	0	1	0	0	0	1	1
43	*Rhantusvermiculatus* Moschulsky, 1860	0	0	0	0	0	0	0	1	2	0	2	12
** Dytiscinae **													
44	*Aciliuscanaliculatus* Nicolai, 1822	0	0	0	0	0	0	0	1	1	0	2	2
45	**Aciliussulcatus* Linnaeus, 1758	0	0	0	0	0	0	0	1	0	0	1	1
46	*Graphoderusaustriacus* Sturm, 1834	1	0	0	0	1	0	0	1	1	0	4	4
47	*Graphoderuscinereus* Linnaeus, 1758	1	1	0	0	0	0	0	1	0	0	3	3
48	*Graphoderuszonatusverrucifer* Sahlberg, 1824	0	0	0	0	0	0	0	1	0	0	1	1
49	*Graphoderuszonatuszonatus* Hoppe, 1795	1	0	0	0	0	0	0	1	1	0	3	3
50	**Dytiscuscircumcinctus* Ahrens, 1811	1	1	0	0	0	0	0	1	0	0	3	3
51	*Dytiscusdauricus* Gebler,1832	1	1	0	1	0	1	1	1	0	0	6	6
52	*Dytiscuslatro* Sharp, 1882	1	0	0	1	0	0	0	1	0	0	3	3
53	**Hydaticusaruspex* Clark, 1864	0	0	0	0	0	0	0	1	0	0	1	1
54	**Hydaticuscontinentalis* Balfour-Browne, 1944	0	0	0	0	0	0	0	1	0	0	1	1
** Hydroporinae **													
55	**Bidessusnasutus* Sharp, 1887	1	0	1	0	0	0	0	0	0	0	2	8
56	**Bidessusunistriatus* Goeze, 1777	0	0	0	1	0	0	0	0	0	0	1	1
57	*Hydroglyphusgeminus* Fabricius, 1792	0	1	1	0	0	0	0	1	0	0	3	16
58	**Hydroglyphushamulatus* Gyllenhal, 1813	1	0	0	0	0	0	0	0	0	0	1	1
59	*Hydroglyphuslicenti* Feng, 1936	0	0	0	0	0	0	0	1	0	0	1	1
60	**Boreonectesaff.emmerichi* Falkenström, 1936	1	0	0	0	0	0	0	0	0	0	1	3
61	*Nebrioporusairumlus* Kolenati, 1845	1	1	1	1	0	0	0	1	1	0	6	32
62	**Nebrioporusassimilis* Paykull, 1798	1	0	0	0	0	0	0	0	0	0	1	1
63	*Nebrioporusdepressus* Fabricius, 1775	1	0	1	0	0	0	0	1	0	0	3	4
64	**Nebrioporushostilis* Sharp, 1884	1	0	0	0	0	0	0	1	0	0	2	3
65	*Oreodytesmongolicus* Brinck, 1943	1	1	1	1	0	1	0	1	1	1	7	40
66	*Oreodytesseptentrionalis* Gyllenhal in C.R. Sahlberg, 1826	1	0	1	1	0	1	0	1	0	1	5	27
67	*Oreodytesshorti* Shaverdo & Fery, 2006	0	1	0	0	0	0	0	1	0	0	2	4
68	*Nectoporussanmarkii* Sahlberg, 1826	1	1	1	1	0	0	0	1	0	0	5	45
69	*Zaitzevhydrusformaster* Zaitzev, 1908	1	0	0	0	0	0	0	1	1	0	3	20
70	*Hydroporusacutangulus* complex Thomson, 1856	1	0	1	0	0	1	1	1	1	0	6	36
71	*Hydroporusangusi* Nilsson, 1990	0	0	0	0	0	0	0	1	0	0	1	1
72	**Hydroporuscrinitisternus* Shaverdo &	0	0	0	0	0	0	0	0	0	1	0	1
73	*Hydroporuselongatulus* Sturm, 1835	0	0	0	0	0	0	0	1	0	0	1	2
74	*Hydroporusfuscipennis* Schaum & Kiesenwetter, 1867	0	0	0	0	0	0	0	1	0	0	1	3
75	*Hydroporusgeniculatus* Thomson, 1856	1	0	0	0	0	0	0	1	1	0	3	9
76	*Hydroporuskabakovi* Fery & Petrov, 2006	0	0	0	0	0	0	0	1	0	0	1	2
77	*Hydroporusmorio* Aubé, 1838	0	0	0	0	0	0	0	1	0	0	1	5
78	*Hydroporusnigellus* Mannerheim, 1853	0	0	0	0	0	0	0	1	0	0	1	3
79	*Hydroporusnotabilis* LeConte, 1850	0	0	0	0	0	0	0	1	0	0	1	6
80	*Hydroporuspalustris* Linnaeus, 1760	0	0	0	0	0	0	0	1	0	0	1	1
81	*Hydroporussibiricus* Sahlberg, 1880	0	0	0	0	0	1	0	1	0	0	2	5
82	*Hydroporussubmuticus* Thomson, 1874	0	0	0	0	0	0	0	1	0	0	1	9
83	*Hydroporusuenoi* Nakane, 1963	0	0	0	0	0	0	0	1	0	0	1	13
84	*Hygrotuscaspius* Wehncke, 1875	0	0	0	0	1	1	0	1	0	0	3	10
85	*Hygrotusconfluens* Fabricius, 1787	0	1	0	0	0	0	0	0	0	0	1	1
86	*Hygrotusenneagrammus* Ahrens, 1833	1	1	1	0	0	0	0	1	0	0	4	9
87	*Hygrotusflaviventris* Motschulsky, 1860	1	1	1	1	1	1	0	1	0	1	7	24
88	*Hygrotusnigrolineatus* Steven in Schönherr, 1808	1	1	0	0	1	0	0	1	0	0	4	6
89	*Hygrotuspectoralis* Motschulsky, 1860	1	0	1	0	0	0	0	1	0	0	3	3
90	*Hygrotusinaequalis* Fabricius, 1777	0	0	1	0	0	0	0	1	1	0	3	8
91	*Hygrotusquinquelineatus* Zetterstedt, 1828	1	0	0	0	0	0	0	1	1	0	3	17
92	*Hygrotuschinensis* Sharp, 1882	0	1	0	0	0	1	0	1	0	0	3	4
93	*Hygrotusimpressopunctatus* Schaller, 1783	1	1	1	1	0	1	0	1	1	0	7	72
94	*Hygrotusmarklini* Gyllenhal, 1813	1	1	0	1	1	1	1	1	1	1	9	53
95	*Hygrotusparallellogrammus* Ahrens, 1812	0	0	0	1	0	0	0	1	0	0	2	3
96	*Hygrotusunguicularis* Crotch, 1874	1	1	1	0	0	1	0	1	0	0	5	31
97	*Hygrotusurgensis* Jakovlev, 1899	0	0	0	0	0	0	0	1	0	0	1	1
98	**Laccornisoblongus* Stephens, 1835	0	0	0	0	0	0	0	1	0	0	1	1
** Laccophilinae **													
99	*Laccophilusbiguttatus* Kirby, 1837	1	1	0	1	0	1	1	1	1	0	7	44
	**Overall species composition**	**45**	**26**	**23**	**21**	**9**	**17**	**16**	**79**	**21**	**5**		

* Sampling points by others DGLB- Depression of Great Lakes Basin; GRB-Gobi River Basin; TRB-Tes River Basin; VLRB-Valley of Lakes River Basin; KhGRB-Khalkh Gol River Basin; KhRB-Kherlen River Basin; ORB-Orkhon River Basin; SRB-Selenge River Basin; ShRB-Shishkhed River Basin; BRB-Bulgan River Basin.

## Supplementary materials

Supplementary material 1 Species list of Dytiscid (Coleoptera, Dytiscidae) of Mongolia Data type: Table data This dataset is made available under the Open Database License (http://opendatacommons.org/licenses/odbl/1.0/). The Open Database License (ODbL) is a license agreement intended to allow users to freely share, modify, and use this Dataset while maintaining this same freedom for others, provided that the original source and author(s) are credited.Davaadorj Enkhnasan, Bazartseren Boldgiv
